# TAVR: A Review of Current Practices and Considerations in Low-Risk Patients

**DOI:** 10.1155/2020/2582938

**Published:** 2020-12-24

**Authors:** Jenna Spears, Yousif Al-Saiegh, David Goldberg, Sina Manthey, Sheldon Goldberg

**Affiliations:** ^1^Department of Medicine, Pennsylvania Hospital, University of Pennsylvania Health System (UPHS), Philadelphia, PA, USA; ^2^Department of Cardiology, Pennsylvania Hospital, University of Pennsylvania Health System (UPHS), Philadelphia, PA, USA

## Abstract

Transcatheter aortic valve replacement (TAVR) is an established treatment for severe, symptomatic, aortic stenosis (AS) in patients of all risk categories and now comprises 12.5% of all aortic valve replacements. TAVR is a less invasive alternative to traditional surgical aortic valve replacement (SAVR), with equivalent or superior outcomes. The use of TAVR has increased rapidly. The success and increase in use of TAVR are a result of advances in technology, greater operator experience, and improved outcomes. Indications have recently expanded to include patients considered to be at low risk for SAVR. While TAVR outcomes have improved, remaining challenges include the management of coexistent coronary artery disease, prevention of periprocedural stroke, and issue of durability. These issues are even more relevant for low-risk, younger patients.

## 1. Introduction

Transcatheter aortic valve replacement (TAVR) has been a rapidly evolving field since the first valve was implanted in an inoperable patient with severe aortic stenosis in 2002, amidst strong early criticism [1]. TAVR has now been performed in over 400,000 patients worldwide [1]. Randomized controlled trials (RCT) have demonstrated the safety and efficacy of TAVR, first in inoperable, and then in high-risk, intermediate, and most recently low-risk patients. The success and rapid evolution of TAVR have grown as a result of advances in technology and operator experience. TAVR faces many challenges, especially surrounding durability in low-risk patients. We review the current status of TAVR with emphasis on patient selection, preprocedural workup, and limitations and challenges which are especially relevant in low-risk patients [2].

## 2. Epidemiology of Aortic Stenosis

Aortic stenosis (AS) is a common valvular disease in developed countries [3]. It is most frequently caused by age-related valvular calcification and less likely rheumatic heart disease [3, 4]. As the population ages, aortic stenosis will become an increasingly significant health burden [5]. The prevalence of aortic stenosis increases with age and affects 2.8% of patients aged 60–74 years and 13.1% in patients 75 years and older, which corresponds to approximately 16.1 million people [5]. The estimated number of patients with severe aortic stenosis is 3.2 million, and approximately one million of them are eligible for TAVR [5]. Of these patients eligible for TAVR, approximately 378,890 are considered to be low risk [5]. If left untreated, severe aortic stenosis is associated with a mortality rate of up to 50%, within 3–5 years after symptom onset [5].

## 3. Evolution of TAVR, and Where We Are Now

Transaortic valve replacement (TAVR) has been studied in patients with severe, symptomatic (NYHA Class II or worse) aortic stenosis of varying perioperative risks. The first TAVR trials were conducted in patients considered to be inoperable [6, 7]. TAVR was superior to standard therapy, which included balloon valvuloplasty in inoperable patients [6, 7]. In high-risk patients, TAVR was noninferior to surgical aortic valve replacement (SAVR) for all-cause mortality [8–10]. However, TAVR was associated with significantly higher rates of major vascular complications and neurological events [8, 9]. TAVR was subsequently studied in intermediate-risk patients and found to be noninferior to SAVR for all-cause mortality and disabling stroke but continued to be associated with more periprocedural major vascular complications and higher rates of significant paravalvular regurgitation [11–14]. Based on these findings, the 2017 AHA/ACC guidelines for the management of severe, symptomatic aortic stenosis were changed [15]. TAVR received an I (*A*) recommendation for both inoperable (with predicted survival of over 1 year) and high-risk patients, and a IIa (B) recommendation for intermediate-risk patients [15].

TAVR in low-risk patients has been studied in recent randomized controlled trials (RCTs) including NOTION, Evolut R Low Risk, and PARTNER III. None of the aforementioned trials studied TAVR for patients with bicuspid aortic stenosis, congenital AS, rheumatic valve disease, or isolated aortic regurgitation [16, 17] [Table 1].

The PARTNER III trial showed that TAVR was superior to SAVR for the primary endpoints of all-cause mortality, stroke, rehospitalization, and new-onset atrial fibrillation at one year [16]. There were no significant differences between SAVR and TAVR for major vascular complications or moderate to severe paravalvular regurgitation [16]. Compared with SAVR, TAVR was associated with a 50% reduction in length of hospital stay, as these patients less frequently required general anesthesia and intensive care unit level care [16]. Given these results, the United States Food and Drug Administration expanded the indications for TAVR to low-risk severe AS patients [20]. Two-year follow-up data in low-risk TAVR patients showed persistent superiority for the combined primary endpoint (death, stroke, or cardiovascular rehospitalization) and rehospitalization alone [2]. Initially, at 1 year, the outcomes of death and stroke strongly favored TAVR; however, this benefit was diminished at two years [2].

A meta-analysis of four RCTs (NOTION, PARTNER III, SURTAVI, and Evolut Low-Risk) comparing TAVR and SAVR outcomes in low-risk patients found that TAVR was associated with a significantly lower risk of all-cause and cardiovascular mortality at one year [21]. The results of this meta-analysis differed significantly from PARTNER III. In the meta-analysis, there was no significant difference in the stroke rate between TAVR and SAVR; TAVR was associated with significantly higher rates of permanent pacemaker implantation and moderate to severe paravalvular leak [21]. This study also showed no significant difference between SAVR and TAVR in the rate of major vascular complications [21]. In this meta-analysis of low-risk patients, the improved all-cause mortality in TAVR compared with SAVR was also reflected in a meta-analysis of patients of all surgical risk categories [22]. However, in the meta-analysis of patients of all risk categories, TAVR was associated with a significantly lower risk of stroke, [22] but significantly increased risk of major vascular complications and permanent pacemaker implantation, compared with SAVR [22]. Follow-up data will be needed to assess long-term results of TAVR compared with SAVR, especially in low-risk patients.

## 4. Patient Selection

Patient selection begins with a careful history and physical exam. Valve anatomy and hemodynamics are then established with transthoracic echocardiography (TTE) [17]. The severity of aortic stenosis is commonly assessed by noninvasive methods such as Doppler TTE but may also be diagnosed during cardiac catheterization [4]. Invasive evaluation is indicated when there is a discrepancy between noninvasive testing and clinical evaluation and the suspicion for significant AS remains high [4].

Severe aortic stenosis is classified by a valve area <1.0 cm^2^ and a peak aortic velocity ≥4.0 m/s with a mean valve gradient ≥40 mm Hg [4]. Variants of classic severe AS, such as low-flow and low-gradient (LFLG) AS are important to consider when evaluating for TAVR. These patients may have concomitant reduced LVEF, with lower peak velocity and gradient than would be anticipated with the severely reduced valve area [4]. The mechanisms of LFLG AS include reduced flow due to LV systolic dysfunction or diminished ventricular volume from a stiff, hypertrophied left ventricle [4]. LFLG AS patients have a higher associated mortality postintervention, as compared with patients with high gradient severe symptomatic AS [4, 17].

## 5. Risk Stratification

The AHA/ACC recommends assessing TAVR perioperative risk with the Society of Thoracic Surgeons Predicted Risk of Mortality (STS PROM) score and an assessment of frailty, comorbidities, and procedural impediments [17].

The STS PROM score predicts the 30-day mortality risk of SAVR and categorizes patients as low to high risk. Patients with <4% predicted mortality are considered low risk, those with 4–8% are intermediate risk, and those with >8% are high risk [17]. Patients with a >50% preoperative risk of mortality and morbidity at 1 year are considered inoperable [17]. Although the STS score was derived from a surgical patient database, it has continued to be applied to TAVR patients, given its use in the original TAVR trials [11, 23].

The STS score has been updated with the 2018 version being the most current [23]. This updated version differs significantly from the previous 2008 version, which was used in the early TAVR trials to assess patient risk [23]. Notably, based on the updated score, 19% of patients from the original TAVR trials would now be reclassified to a lower risk category [23]. This complicates risk stratification and should be considered when evaluating patients for TAVR. [23].

The STS score is also limited in its ability to predict 30-day and 1-year TAVR mortality [24–27]. The STS score overestimates the 30-day mortality in TAVR and does not accurately reflect the impact of comorbidities on TAVR [23, 24]. The STS score overpredicts diabetics' mortality risk in TAVR, while the opposite is true for patients with atrial fibrillation [23]. TAVR-specific prognostic scores are not routinely used in preoperative evaluation but may have a role in future practice [26]. Two examples of these are the TAVI_2_SCORe and the STS Transcatheter Valve Therapy Registry, which have been shown to be better predictors of mortality compared with the STS score [28, 29]. Preoperative TAVR risk assessment should be done by a multidisciplinary heart team. This assessment may include traditional risk scores; however, their limitations should be recognized [26] [Table 2].

## 6. Preprocedural Workup

Preprocedural workup is essential to reduce procedural complications and to risk-stratify patients. Patients should be assessed for major cardiovascular and noncardiovascular comorbidities prior to TAVR [17].

The initial assessment of aortic stenosis is completed with TTE which evaluates the severity of stenosis, leaflet motion, annular size, and degree of calcification [17]. The severity of AS is classified based on the calculated aortic valve area (AVA) and the mean transaortic gradient [31] [Table 3]. The role of transesophageal echocardiography (TEE) for TAVR preoperative assessment has been diminished by the advent of CT [17]. TTE can be supplemented with ECG-gated multidetector computed tomography (MDCT) during preprocedural TAVR planning, to provide a three-dimensional anatomical assessment [17].

Coronary angiography is currently the standard practice to evaluate for CAD, prior to TAVR [32]. However, coronary computed tomography angiography (CCTA) has been increasingly utilized [32]. CCTA allows patients to avoid invasive angiography and has an excellent negative predictive value (NPV) [32]. Conversely, the presence of calcified vessels or prior stents leads to false-positive results and limits CCTA's ability to assess the severity of coronary lesions [32]. Coronary angiography is performed to confirm the presence and severity of CAD, after a positive CCTA scan [32]. Although CCTA is a convenient alternative for coronary angiography, ensuring proper patient selection with low pretest probability for coronary disease is important [32]. Preoperative CCTA may decrease the number of invasive coronary angiograms in low-risk patients [32].

A CTA of the chest, abdomen, and pelvis is done to identify peripheral vascular disease. Although transfemoral access is preferred for TAVR, alternatives such as transapical, transaortic, or subclavian approaches are occasionally pursued based on peripheral vascular suitability [33]. Transfemoral access has an associated mortality benefit over other approaches and allows for a shorter hospital stay [34].

## 7. Procedural Sedation and Minimalist Approach

TAVR was historically primarily done under general anesthesia (GA) with endotracheal intubation and periprocedural TEE [17]. TAVR centers are increasingly using a minimalist approach with conscious sedation (CS) instead of GA, although there is still significant variation in CS use between hospitals [35]. A recent study of 120,000 patients from the Transcatheter Valve Therapy Registry showed an increase in the proportion of transfemoral TAVR cases done under CS, from 33% in 2016 to 64% in 2019 [35]. The use of conscious sedation was associated with lower in-hospital and 30-day mortality compared with GA [35]. Avoiding GA with intubation is associated with shorter procedure time, fluoroscopy time, ICU length of stay, and hospital length of stay and decreases the need for inotropic support [36, 37]. TAVR with CS has comparable rates of periprocedural complications such as PPM implantation, MI, stroke, vascular complications, and residual PVL as GA [37].

There are individual patient factors that favor the use of GA, such as morbid obesity, obstructive sleep apnea, an inability to lay flat during the procedure, or the need for an alternate, nontransfemoral access site [35, 38]. Minimally invasive approaches may play a larger role for higher-risk patients with multiple comorbidities, chronic obstructive lung disease, or difficult airways, as GA is associated with more complications in this patient group [39, 40]. The use of CS may further improve the short-term mortality of TAVR, which is of special importance in low-risk patients. Low-risk patients are more likely to be candidates for minimalist TAVR with CS. The convenience, short-term mortality benefit, and less invasive nature of minimalist TAVR will likely be additional factors that influence a low-risk patient's decision between TAVR and SAVR.

## 8. Challenges Facing TAVR in Low-Risk Patients

Many challenges remain surrounding TAVR in low-risk patients. Comparative TAVR and SAVR outcomes in the low-risk TAVR trials are listed in Table 4.

### 8.1. Vascular Complications

The rate of major vascular complications in TAVR has decreased but still occur in >4% of procedures [6–8, 11, 41, 42]. This is the result of improved operator technique, reduced delivery system sheath size, and vascular access closure devices [41]. Early TAVR required large (20 to 24 Fr) sheaths, which more often necessitated transapical or transaortic access [42, 43]. Current generation TAVR devices feature lower profile (14-Fr to 16-Fr) delivery systems [42]. The smaller size allows for transfemoral approach access, more precise valve positioning, and delivery while reducing the risks of major vascular complications [42, 44]. The increased use of direct ultrasound guidance and micropuncture technique has substantially reduced femoral vascular complications [45]. Transfemoral access is preferable to other access methods, as it is associated with mortality benefit, shorter hospital stays, and faster recovery [13].

### 8.2. Coronary Artery Disease

Almost half of TAVR candidates have coexisting coronary artery disease (CAD), with many having multivessel CAD [32]. Prosthetic valve struts can obstruct coronary ostia and therefore complicate accessing the coronaries during percutaneous coronary intervention (PCI) [32]. Therefore, a TAVR patient's future ability to successfully undergo PCI may be affected by the presence of a prosthetic valve.

Ongoing trials are evaluating PCI in patients with stable CAD undergoing TAVR [32]. Common practice is to revascularize proximal-mid coronary lesions pre-TAVR. [32]. Pre-TAVR staged PCI is more commonly done than a combined procedure [32]. Overall mortality has not been shown to be affected by the timing of PCI [32]. However, patients who underwent PCI within 30 days before TAVR had more bleeding and minor vascular complications after TAVR compared with patients who underwent PCI >30 days prior to TAVR [32].

Aggressive management of modifiable cardiac risk factors, through smoking cessation, weight loss, regular physical activity, and use of a moderate or high-intensity statin, can decrease the incidence and progression of CAD and is especially important in TAVR patients [46, 47].

### 8.3. Paravalvular Regurgitation

Paravalvular regurgitation (PVR) is a complication that occurs due to incomplete apposition between the aortic annulus and the device [48]. Echocardiography can identify PVR after TAVR, although invasive hemodynamics and cine-angiography can also be utilized [49]. Risk factors for PVR include valve calcification, leaflet asymmetry, prosthesis malposition, or undersizing and the use of self-expanding valves [50]. Self-expanding valves exert less radial force than their balloon-expandable counterparts [50]. Annular calcification has a larger effect on the final figuration of self-expanding valves, and therefore they are more often underexpanded or eccentrically shaped. [50]. Although balloon-expandable devices can generate higher forces to overcome severe calcification, this can lead to annular rupture [50].

The severity and acuity of valvular regurgitation play a role in patient outcomes after TAVR [51]. Moderate and severe PVR are independent predictors of early and late mortality, while the significance of mild PVR is unclear [51]. Moderate to severe PVR occurs in 3.7–5.3% of intermediate-risk patients and in 0.6–3.5% of low-risk patients [11, 12, 16, 18]. Mild PVR occurs in 23–36% of intermediate-risk patients and 30–36% of low-risk patients [11, 12, 16, 18]. After TAVR, the acute onset of moderate to severe aortic regurgitation (AR) is associated with higher mortality and should be promptly managed to decrease the regurgitant volume. [51]. Therapeutic strategies include balloon postdilatation, leak closure with vascular plugs, implantation of a second valve, or even surgical removal of the prosthetic valve [51]. After TAVR, chronic AR refers to the presence of moderate to severe regurgitation, which is not worse compared with the degree of AR pre-TAVR [51]. Chronic AR does not have the same high mortality as acute AR but should be closely monitored [51].

Although SAVR has historically had a lower rate of moderate or severe PVR than TAVR, PARTNER III TAVR patients had comparable rates to SAVR patients at 30 days [Table 2] [16]. Decreasing the rate of significant PVR in low-risk patients is especially important as it directly impacts early and long-term mortality. The findings from PARTNER III will likely influence preprocedural planning, as the SAPIEN 3 valve may improve TAVR outcomes for low-risk patients who are at high-risk of PVR based on anatomic factors.

The Evolut Low-Risk Trial PVR outcomes were less promising, as TAVR patients had a significantly greater incidence of moderate to severe PVR (3.5%) than SAVR (0.5%) [18]. These findings are likely related to the fact that PARTNER III features a balloon-expandable valve (SAPIEN 3), while Evolut featured a self-expanding valve (CoreValve, Evolut R, and Evolut PRO).

Current generation TAVR has decreased the incidence of significant valvular regurgitation at 30 days, due to improved prosthesis design [44]. New-generation valves have design features which minimize PVR, including the external fabric skirt of SAPIEN 3 valve or the external sealing system of the Evolut PRO valve [51]. Although self-expanding Evolut valves were associated with increased PVR, they also had less patient-prosthesis mismatch [18]. Patient-prosthesis mismatch is associated with higher incidences of perioperative stroke, renal failure, and lack of ventricular regression after TAVR [52]. Severe patient-prosthesis mismatch is also felt to have an impact on mortality, although this is controversial in studies [52]. Only 1.8% of TAVR patients in the Evolut Low-Risk Trial had severe mismatch, compared with 8.2% of SAVR patients [18]. The patients in the Evolut Trial also had less mismatch than the patients in the PARTNER III, where 4.3% of TAVR patients developed severe mismatch [16, 18]. Risk factors for mismatch include older age, female sex, diabetes, renal failure, and higher surgical risk scores [52]. These risk factors occurred at similar rates in the Evolut and PARTNER III patient groups, which may suggest that the difference in outcomes is due to valve characteristics. Design features that minimize PVR may worsen conduction system complications and patient-prosthesis mismatch.

### 8.4. Permanent Pacemaker Implantation

During TAVR implantation, trauma to the conduction system, namely, the bundle of His and the left bundle branch, can occur and result in complete heart block or left bundle branch block (LBBB). Implantation of a permanent pacemaker (PPM) is most often necessitated as a result of new high-degree AV block and in a minority of patients for sick sinus syndrome [53].

TAVR is associated with significantly more PPM implantation than SAVR, in patients of all-risk categories. [21, 22]. Complications of new PPM implantation were similar in recent low-risk TAVR trials to the those in the previous studies in higher-risk patients [54]. The Evolut Low-Risk TAVR Trial with self-expanding valves also showed that significantly more low-risk TAVR patients underwent postoperative permanent pacemaker (PPM) implantation than SAVR patients (17.4% vs. 6.1%, respectively) [18]. The PARTNER III Trial had 6.6% of TAVR patients requiring PPM, which was found to not be significantly different compared with SAVR (at 4.1%) [16]. However, significantly more patients in the TAVR cohort developed new LBBB than SAVR patients (22% compared to 8%, respectively) [16].

Factors that impact the need for PPM in TAVR are preexisting conduction abnormalities, calcification of the LVOT, balloon valvuloplasty, and depth of THV implantation [53, 55]. Balloon-expandable THV are associated with a lower risk of PPM implantation than self-expanding valves [37, 53, 55, 56]. This is felt to be due to the increased radial force on the LVOT by self-expanding valves [54]. For example, an average of 25.8% of TAVR cases with the self-expanding CoreValve is associated with new PPM, while the average rates in TAVR with balloon-expanding SAPIEN valve are much lower at 6.5% [53]. The type of THV also plays a role, as seen with the higher rates of PPM with newer generation SAPIEN 3 THV, compared with the previous generation SAPIEN XT [56, 57]. The higher complication rate in SAPIEN 3 valves are attributed to the increased length, implantation height, and radial force exerted by its fabric skirt [54, 57]. Preoperative TAVR evaluation should include assessment for patient risk factors such as baseline conduction disturbances and LVOT calcification to assist procedural planning and to minimize the risk of PPM implantation [55].

Despite improvements in many of the complications of TAVR, the incidence of new conduction system disease and PPM implantation have remained steady or increased [56]. Although PPM implantation is more often required in higher-risk patients, the implications of a PPM are especially significant in low-risk TAVR patients [58]. Understanding the patient and procedural factors that predict postoperative PPM insertion are important when counseling low-risk patients about the possible need for PPM [55]. Conduction abnormalities that occur as a complication of TAVR are transient in a considerable number of patients [54]. Cohort studies that included a variety of TAVR prostheses have shown that long-term pacemaker dependency ranged from 27% to 68% of patients with PPM [54]. Therefore, the optimal timing of PPM implantation and long-term benefit of PPM are unclear [55]. To avoid unnecessary PPM, it may be reasonable to have patients undergo a period of rhythm monitoring after TAVR before PPM implantation. Many patients with severe aortic stenosis also have left ventricular hypertrophy and underlying heart failure. Conduction system disease and the need for pacing in these patients can lead to new heart failure or worsen existing cardiac function. On average, most PPMs typically last 10–15 years [59, 60]. The lifetime of a pacemaker is important to be considered, especially in low-risk patients. Patients should be counseled that they may require PPM exchange in the future, which is another procedure that carries risks of complications.

Studies of the prognostic consequences of PPM implantation in TAVR have had conflicting findings. Some studies have shown that PPM has no negative impact on 1-year mortality, while results from the TVT registry have shown that PPM implantation is associated with increased mortality [53, 54, 58]. A recent study in intermediate-risk patients evaluated the incidence of new LBBB after TAVR and found that 15.2% of TAVR patients developed new LBBB. This did significantly increase the all-cause and cardiovascular mortality, rehospitalization, PPM implantation and decreased LV function at 2 years [56]. This would suggest that PPM is associated with at least cardiovascular morbidity, which likely puts patients at risk of long-term increased mortality. Further studies are needed to define the impact that PPM has on cardiovascular morbidity and mortality and to establish the optimal timing of PPM implantation to avoid placement in patients with transient conduction abnormalities.

### 8.5. Stroke and the Role of Cerebral Protection

Stroke is an important cause of morbidity and mortality in TAVR [61]. The incidence of stroke after TAVR varies considerably [61]. Stroke complicates 2.7 to 5.5% of cases at 30 days but is underestimated in many trials [61, 62]. In PARTNER III, only 1.2% of low-risk TAVR patients had a major stroke at 1 year, compared with 3.1% of SAVR patients [16]. This was significantly lower than in the previous trials in higher-risk patients [8, 11, 12, 16]. However, at 2 years, TAVR was no longer statistically superior to SAVR [2]. Many studies report only major clinical strokes and do not include sensitive assessments of stroke such as evaluation by neurologists or imaging [61, 62]. Several studies have shown that, with routine MRI screening, the incidence of stroke after TAVR is 9–28% at 30 days [61, 63]. The Neurological Research Consortium has made recommendations to make evaluation of neurological endpoints more uniform [64]. In CEP studies, the recommended early efficacy endpoints are overt CNS injury, CNS infarction and hemorrhage, neurological dysfunction (TIA), MRI total lesion volume, and cognitive change [64]. They recommend that all eligible patients receive a baseline MRI for subtraction against the postprocedure MRI [65].

Approximately half of the strokes that occur after TAVR are periprocedural (within 48 hours of TAVR) and are embolic in nature [62, 66, 67]. Periprocedural stroke increases the 30-day mortality by 4- to 6-fold [62, 68, 69]. Cerebral embolic protection devices (CEPD) prevent embolization of debris to the brain during TAVR and may play a role in preventing periprocedural stroke [61, 67]. The routine use of CEPD for stroke prevention in TAVR has been controversial. The utility of CEPD in TAVR was highlighted in the SENTINEL Trial, where embolic debris was captured in 99% of patients [67, 70]. When the debris was analyzed, it not only consisted of the anticipated thrombus and calcium, but also included foreign material (35% of patients), arterial wall, valve tissue, and myocardium [67, 70]. This study showed no clinically significant reduction in stroke; however, there was a 42% reduction in new lesion volume on diffusion-weighted MRI (DW MRI) in the CEPD group compared with that in the unprotected group [67]. Silent infarcts are evident only on imaging and have no associated focal neurological dysfunction attributed to them [61]. However, silent infarcts are associated with an increased risk of dementia and independently increase the risk of cognitive decline and future clinical strokes by 2- to 4-fold [62, 65]. CEP devices may play a role in preventing silent infarcts, which the majority of patients develop after TAVR [61, 67].

Despite the high frequency of embolization of debris in TAVR, CEPDs are not commonly used. Current RCTs have significant limitations in study size and have failed to show any significant reduction in stroke or mortality with CEPD [61]. Meta-analysis has also shown reductions in death and stroke with CEPD; however, these results were not statistically significant [61]. Given the lack of statistically significant results, the routine use of CEPDs in TAVR is not supported in the guidelines [61]. Despite the lack of widespread use, CEPDs have rapidly evolved with TAVR. New CEPDs cover all three aortic cerebral branches, as seen with TriGUARD 3, and offer more vascular protection than the previous devices [61, 62].

Registries have shown that operator experience and increasing site volume were associated with better outcomes in regards to mortality, vascular complication, and bleeding but not for stroke [66]. The role of CEPD in TAVR is yet to be determined by further RCTs that are adequately powered and include sensitive assessments of stroke [61]. The argument for the use of CEPD is even stronger in younger, low-risk patients who have more time to develop long-term cognitive effects of silent infarcts.

### 8.6. Durability

A major challenge facing TAVR in low-risk patients is a limited valve lifespan, as these patients are usually younger with less comorbidities and are more likely to require repeat procedures. The mean age of patients in the recent low-risk TAVR trial was 73 years versus 82 years in the intermediate-risk TAVR trials [11, 16]. There are no explicit guidelines advising which age of patients derives lasting benefit from TAVR implantation.

While the longevity of surgical bioprosthetic valves is well studied, there is a lack of similar data for TAVR valves. Transcatheter bioprosthetic valves can degenerate similar to surgical bioprosthetic valves; however, the longevity of TAVR valves may be further limited by the trauma and mechanical stress to the valve during the preparation, dilatation, or positioning of the valve. Data from early TAVR studies cannot be applied to current low-risk patients. This is primarily due to the rapid turnover of prosthetic valves and the differences in the patient population. In studies of surgical bioprosthetic valves, younger age at implantation has been shown to be associated with increased structural valve degeneration (SVD), especially in patients under the age of 60 [71, 72].

SVD is an irreversible intrinsic change, such as leaflet tear or calcification, that leads to deterioration and/or dysfunction of the valve [73]. SVD is an important etiology of bioprosthetic valve failure (BVF) as it can result in eventual stenosis or regurgitation [73]. Meta-analyses in surgical bioprosthetic valves have shown SVD begins 8 years after implantation, with further substantial increased SVD after 10 years [74]. The incidence of SVD after TAVR varies in the literature, from <5% to 10% at 1 year, 12–20% at 5 years, and approximately 13–23% at 8 years [74–76]. Other sources of BVF include nonstructural valve dysfunction, valve thrombosis, and endocarditis. It is important to differentiate the source of valve failure as these other sources may be reversible [73].

When approaching TAVR in low-risk patients, the limited evidence of valve durability has to be taken into account. Patients must be counseled on the potential need for repeat procedures.

## 9. Conclusion and Future Directions

TAVR has rapidly evolved since its inception and is now indicated for severe, symptomatic aortic stenosis in patients of all risk categories. There are clear advantages to TAVR, as the focus is placed on minimally invasive procedures to reduce complications and length of stay. TAVR now accounts for 12.5% of all aortic valve replacements [77]. There is still uncertainty regarding the optimal management of coexisting CAD, the prevention of periprocedural stroke, and the durability of TAVR. Further studies are needed to develop and assess the efficacy of TAVR-specific risk scores, to better characterize patient risk preoperatively. Other areas of interest will include defining the durability, long-term morbidity, and mortality associated with transaortic bioprosthetic valves in low-risk patients. The effects of permanent pacemaker implantation and patient-prosthesis mismatch on long-term morbidity and mortality are still being studied and are of particular importance to low-risk patients. Certain trade-offs exist, and individualized preoperative evaluation is needed to determine the best choice of prosthesis. Patient preference may be for TAVR; however, in select low-risk patients, a surgical mechanical valve may be a better option. Understanding the long-term risks of TAVR is essential to counseling low-risk patients about their choice of procedure.

Lastly, certain aortic valve disease states were excluded from TAVR trials, including moderate aortic stenosis, aortic insufficiency, and bicuspid aortic valves. Therefore, TAVR is not indicated in these patients. The management of these patients is a continued challenge and requires further investigation. Future trials in these patients may further expand the indications of TAVR.

## Figures and Tables

**Table 1:** Low-risk TAVR trials [16, 18, 19].

	Notion	Evolut Low-Risk Trial	PARTNER III
Valve type	Self-expanding	Self-expanding	Balloon-expandable

Number of patients	280	1200	1328

Average age	79.1 years	74 years	73 years

Inclusion STS PROM score	N/A (81.8% of patients were at low-risk, with a STS<4)	<3	<4

Mean STS PROM score	3.0%	1.9%	1.9%

Inclusion Criteria	(i) Patients ≥70 years of age with severe degenerative AS referred for SAVR and also candidates for TAVR were eligible for inclusion regardless of their predicted risk of death after surgery. (ii) Severe AS was defined as an effective orifice area <1 cm^2^ or indexed for body surface area <0.6 cm^2^/m^2^ and a mean AV gradient >40 mm Hg or peak systolic velocity >4 m/s. (iii) Symptomatic patients had to have dyspnea, NYHA class II or higher, angina pectoris, or cardiac syncope to qualify for the trial. (iv) Asymptomatic patients could be included if they had left ventricular posterior wall thickness ≥17 mm, decreasing LVEF, or new-onset AF. (v) Eligible patients were expected to survive for more than 1 year.	(A) Severe aortic stenosis: (a) for symptomatic patients, AVA ≤1.0 cm^2^ (or AVA index of ≤0.6 cm^2^/m^2^) or mean gradient ≥40 mmHg or maximal AV velocity ≥4.0 m/sec by TTE at rest. (b) For asymptomatic patients, (i) very severe AS with an AVA of ≤1.0 cm^2^ (or AVA index of ≤0.6 cm^2^/m^2^) and maximal aortic velocity ≥5.0 m/sec, or mean gradient ≥60 mmHg by TTE at rest or (ii) AVA of ≤1.0 cm^2^ (or AVA index of ≤0.6 cm^2^/m^2^) and a mean gradient ≥40 mmHg or maximal AV velocity ≥4.0 m/sec by TTE at rest and an exercise tolerance test that demonstrates a limited exercise capacity, abnormal BP response or arrhythmia or (iii) AVA of ≤1.0 cm^2^ (or AVA index of ≤0.6 cm2/m2) and mean gradient ≥40 mmHg or maximal AV velocity ≥4.0 m/sec by TTE at rest and LVEF <50%. (B) The patient is considered low risk for surgery, with a predicted risk of mortality for surgery <3% at 30 days per multidisciplinary local heart team assessment. C) Agreement that the patient will return for all required postprocedure follow-up visits.	(A) Severe calcific AS with AVA ≤1.0 cm^2^ or AVA index ≤0.6 cm^2^/m^2^ and Jet velocity ≥4.0 m/s or mean gradient ≥40 mmHg. (B) NYHA functional class ≥2 or exercise tolerance test that demonstrates a limited exercise capacity, abnormal BP response or arrhythmia or asymptomatic with LVEF <50% (echo must be within the 90 days prior to randomization). (C) Heart team agrees the patient has a low risk of operative mortality and an STS < 4. D) The patient has provided written informed consent.

Selected exclusion criteria	(i) Other severe valvular disease or CAD requiring intervention. (ii) Previous cardiac surgery. (iii) Stroke or MI within 30 days. (iv) Renal failure requiring dialysis. (v) Pulmonary failure with forced expiratory volume within 1 second (FEV_1_) <40% of expected.	(i) Bicuspid AV. (ii) Preexisting prosthetic heart valve (iii) Candidates for mechanical valves. (iv) Valvular disease (severe MR, severe TR, ≥moderate MS) that is amenable to surgery or repair. (v) Unprotected left main coronary artery and/or multivessel coronary disease with SYNTAX score >22. (vi) Stroke or TIA within 2 months, MI within 30 days. (vii) Estimated life expectancy under 2 years.	(i) AV that is bicuspid, unicuspid, or noncalcified. (ii) Severe MR, severe AR, or ≥ moderate MS. (iii) Preexisting mechanical or bioprosthetic valve (not including mitral ring). (iv) LVEF < 30%. (v) unprotected left main coronary artery, SYNTAX score >32 (in absence of prior revascularization), no ability for optimal coronary revascularization. (vi) Stroke or TIA within 90 days. (vii) MI within 30 days. (viii) Renal insufficiency (eGFR< 30 mL/min) or requiring renal replacement therapy. (ix) Severe lung disease with FEV_1_ < 50% of predicted or currently on home oxygen. (x) Significant frailty. (xi) History of cirrhosis or active liver disease. (xii) Estimated life expectancy under 2 years.

Endpoint	All-cause mortality, stroke and MI at one year.	All-cause mortality or disabling stroke at 2 years.	All-cause mortality, stroke, and rehospitalization at 1 year

Result	No significant difference between TAVR and SAVR.	TAVR noninferior to SAVR.	TAVR superior to SAVR, with significantly lower rate of death, stroke, or rehospitalizations.

AS = aortic stenosis; AV = aortic valve; AVA = aortic valve area; AR = aortic regurgitation; CAD= coronary artery disease; eGFR = estimated glomerular filtration rate; FEV_1_ = forced expiratory volume within 1 second; LVEF = left ventricular ejection fraction; MI = myocardial infarction; MR = mitral regurgitation; MS = mitral stenosis; STS PROM Score = Society of Thoracic Surgeons Predicted Risk of Mortality Score; SYNTAX score = synergy between percutaneous coronary intervention with taxus and cardiac surgery; TAVR = transcatheter aortic valve replacement; THV = transcatheter heart valve; TIA = transient ischemic attack; TTE = transthoracic echocardiogram; TR = tricuspid regurgitation.

**Table 2:** Comparison of factors used in SAVR and TAVR risk scores [28, 30].

	STS score	EuroSCORE II	TAVI_2_SCORe
Peripheral vascular disease	Yes	Yes	No
Renal failure	Yes	Yes	Yes
Dialysis	Yes	No	No
Neurological dysfunction	Yes	Yes	No
Diabetes	Yes	No	No
Atrial fibrillation	Yes	No	No
COPD	Yes	Yes	No
NYHA class	Yes	Yes	No
LVEF	Yes	Yes	Yes
CAD	Yes	No	No
Pulmonary hypertension	No	Yes	No
Others			Porcelain thoracic aorta, recent myocardial infarction (within 90 days), anemia (<10 g/dl), male sex, critical aortic valve stenosis (mean gradient ≥70 mmHg), age (>85 years)

SAVR = surgical aortic valve replacement; TAVR = transcatheter aortic valve replacement; COPD = chronic obstructive pulmonary disease; NYHA = New York Heart Association Class; LVEF = left ventricular ejection fraction; CAD = coronary artery disease.

**Table 3:** Aortic stenosis severity [4, 31].

	Aortic valve area (cm^2^)	Mean transaortic pressure gradient (mmHg)	Maximum aortic velocity (m/s)
Normal	3.0–4.0	—	—
Mild	1.6–2.0	<25	2.5–3.0
Moderate	1.1–1.5	25–40	3.1–4.0
Severe	≤1.0	>40	>4.0

**Table 4:** Comparison of outcomes between TAVR and SAVR patients in Low-Risk TAVR Trials.

	PARTNER III	Evolut Low-Risk	NOTION^*∗*^
All-cause and CV-mortality at 1 year	TAVR: 1.0 %	TAVR: 2.4%	TAVR: 4.9%
	SAVR: 2.5%	SAVR: 3.0%	SAVR: 7.5%

Disabling stroke at 1 year	TAVR: 0.2%	TAVR: 0.8%	
	SAVR: 0.9%	SAVR: 2.4%	TAVR: 2.9%
Nondisabling stroke at 1 year	TAVR: 1.0%	TAVR: 3.4%	SAVR: 4.6%
	SAVR: 2.2%	SAVR: 2.2%	

TIA at 1 year	TAVR: 1.0%	TAVR: 1.7%	TAVR: 2.1%
	SAVR: 1.1%	SAVR: 1.8%	SAVR: 1.6%

Major vascular complications at 30 days	TAVR: 2.2%	TAVR: 3.8%	TAVR: 5.6%
	SAVR: 1.5%	SAVR: 3.2%	SAVR: 1.5%

AKI (stage II or III) (in 30 days)	TAVR: 0.4%	TAVR: 0.9%	TAVR: 0.7%
	SAVR: 1.8%	SAVR: 2.8%	SAVR: 6.7%

New-onset AF (at 1 year)	TAVR: 7.0%	TAVR: 9.8%	TAVR: 21.2%
	SAVR: 40.9%	SAVR: 38.3%	SAVR: 59.4%

New PPM implantation at 1 year	TAVR: 7.5%	TAVR: 19.4%	TAVR: 38.0%
	SAVR: 5.5%	SAVR: 6.7%	SAVR: 2.4%

Coronary artery obstruction requiring intervention at 1 year	TAVR: 0.2%	TAVR: 0.9%	No TAVR-treated patient required PCI during the procedure; 1 SAVR patient required concomitant coronary artery bypass resulting from a right coronary ostium lesion
	SAVR: 0.7%	SAVR: 0.4%	

MI at 1 year	TAVR: 1.2%	TAVR: 1.7%	TAVR: 3.5%
	SAVR: 2.2%	SAVR: 1.6%	SAVR: 6.0%

Valve thrombosis at 1 year	TAVR: 1.0%	TAVR: 0.2%	Not reported
	SAVR: 0.2%	SAVR: 0.3%	

PVL (≥moderate) at 1 year	TAVR: 0.6%	TAVR: 3.5%	TAVR: 15.7%
	SAVR: 0.5%	SAVR: 0.5%	SAVR: 17.7%

Patient-prosthesis mismatch (30 days)	TAVR moderate: 29.8%	TAVR moderate: 5.0%	Not reported
	SAVR moderate: 23.3%	SAVR moderate: 15.7%	
	TAVR severe: 4.3%	TAVR severe: 1.8%	
	SAVR severe: 6.3%	SAVR severe: 8.2%	

Rehospitalization at 1 year (valve or procedure related, including CHF)	TAVR: 7.3%	TAVR: 3.2%	Not reported
	SAVR: 11.0%	SAVR: 6.5% (CHF rehospitalization only)	

AF = atrial fibrillation; AKI = acute kidney injury; CHF = congestive heart failure; CV = cardiovascular; MI = myocardial infarction; PCI = percutaneous coronary intervention; PPM = permanent pacemaker; PVL = paravalvular leak; TIA = transient ischemic attack. ^*∗*^Although NOTION studied patients of all surgical risks; 81.8% of patients were considered low-risk (STS<4%).

## Data Availability

The data underlying this article are available within the article.

## Abbreviations

ACC: American College of Cardiology
AHA: American Heart Association
AR: Aortic regurgitation
AS: Aortic stenosis
AVA: Aortic valve area
BVF: Bioprosthetic valve failure
CAD: Coronary artery disease
CEPD: Cerebral embolic protection device
CABG: Coronary artery bypass graft
CT Scan: Computed tomography scan
CCTA: Coronary computed tomography angiography
CTA: Computed tomography angiography
DW MRI: Diffusion-weighted magnetic resonance imaging
EuroSCORE II: European System for Cardiac Operative Risk Evaluation
GA: General anesthesia
LBBB: Left bundle branch block
LFLG: Low-flow low-gradient
LVEF: Left ventricular ejection fraction
LVOT: Left ventricular outflow tract
MDCT: Multidimension computed tomography
MI: Myocardial infarction
NPV: Negative predictive value
NYHA: New York Heart Association
PVR: Paravalvular regurgitation
PCI: Percutaneous coronary intervention
PPM: Permanent pacemaker
RCT: Randomized controlled trial
SAVR: Surgical aortic valve replacement
STS PROM Score: Society of Thoracic Surgeons Predicted Risk of Mortality Score
SVD: Structural valve degeneration
TAVI: Transcatheter aortic valve implantation
TAVR: Transcatheter aortic valve replacement
THV: Transcatheter heart valve
TEE: Transesophageal echocardiogram
TIA: Transient ischemic attack
TTE: Transthoracic echocardiogram.
